# Supplementary material for: Efficacy of a Turkey Herpesvirus Vectored Newcastle Disease Vaccine against Genotype VII.1.1 Virus: Challenge Route Affects Shedding Pattern
Source: Vaccines (Basel). 2021 Jan 11;9(1):37. doi: 10.3390/vaccines9010037 (PMC7826937; doi:10.3390/vaccines9010037)
Supplement: Supplementary file 1 [file vaccines-09-00037-s001.pdf]

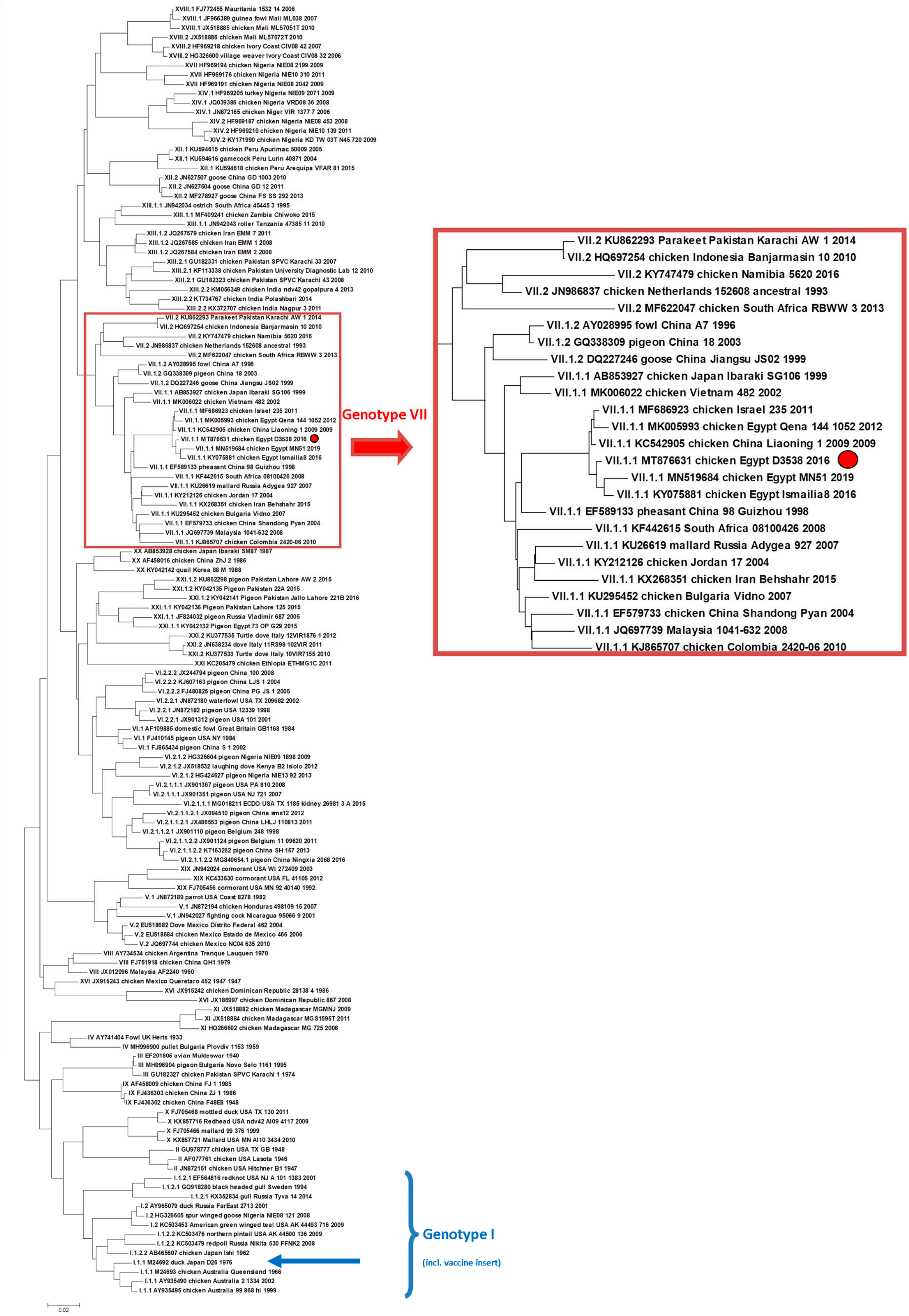

Figure S1. Phylogenetic tree showing the genetic relatedness of challenge virus used in the study to the vaccine insert and other Genotype VII NDV strains based on full fusion (F) gene nucleotide sequences. Sequences were aligned using Kimura 2-parameters method and tree was constructed using the Neighbour-joining method (Mega 7 software). Representative strains and genotyping is according to Dimitrov et al. (2019) [1], additional sequences in genotype VII.1.1 were obtained from

the GenBank. ← symbol indicates the donor strain of the recombinant turkey herpesvirus-Newcastle disease virus vaccine insert (Genotype I), ● symbol indicates the challenge virus (Genotype VII.1.1). Genotype VII branch is magnified from the phylogenetic tree on the right panel. Accession numbers to the nucleotide sequences, year and country of origin are shown for each strain.

Table S1. Pre-challenge serological results of vaccinated chickens submitted to challenge by subgroups.

**Method**	**Age at sampling & challenge (day)**	**20**		**28**	
	**Challenge Route**	**Intra-muscular**	**Intra-nasal**	**Intra-muscular**	**Intra-nasal**
**IDScreen® ND Indirect ELISA**	**ELISA titer** mean ± STD	**4439** ± 2138	**3834** ± 2264	**8537** ± 2543	**8678** ± 1896
	Mann-Whitney test result	*p =0.209*		*p = 0.860*	
**Haemagglutination Inhibition test**	**Log_2 HI titer** mean ± STD	**1.3** ± 0.7	**1.3** ± 0.8	**2.5** ± 0.7	**2.5** ± 0.6
	Mann-Whitney test result	*p = 0.954*		*p = 0.886*	

Scheme 20. in each subgroup). Serum samples were collected from each chicken on the day of challenge to verify the homogeneity of immune response to vaccination between the two subgroups challenged at the same age (Mann-Whitney test was used for comparison).

1. Dimitrov, K.M.; Abolnik, C.; Afonso, C.L.; Albina, E.; Bahl, J.; Berg, M.; Briand, F.-X.; Brown, I.H.; Choi, K.-S.; Chvala, I.; et al. Updated unified phylogenetic classification system and revised nomenclature for Newcastle disease virus. *Infect. Genet. Evol.* **2019**, *74*, 103917, doi:10.1016/j.meegid.2019.103917.
